# A248 ETHANOL-INDUCED MUCOSAL IRRITATION DISRUPTS LOCAL AND SYSTEMIC PROTECTIVE EFFECTS OF NON-INVASIVE TRANSCUTANEOUS AURICULAR VAGUS NERVE STIMULATION (TAVNS)

**DOI:** 10.1093/jcag/gwae059.248

**Published:** 2025-02-10

**Authors:** F Hesampour, C N Bernstein, J Ghia

**Affiliations:** Immunology, University of Manitoba Max Rady College of Medicine, Winnipeg, MB, Canada; University of Manitoba Max Rady College of Medicine, Winnipeg, MB, Canada; Immunology, University of Manitoba Max Rady College of Medicine, Winnipeg, MB, Canada

## Abstract

**Background:**

The integrity of the mucosal barrier is vital for gastrointestinal homeostasis. Previously, we demonstrated that non-invasive transcutaneous auricular vagus nerve stimulation (taVNS) decreases inflammatory and pro-apoptotic markers while increasing anti-inflammatory markers in homeostatic conditions. However, its effectiveness in case of slight mucosal irritation remains unclear.

**Aims:**

To assess whether slight mucosal irritation affects taVNS efficacy in modulating pro- and anti-inflammatory cytokines and apoptosis-related markers in the colon and spleen.

**Methods:**

Male C57BL/6 mice were given a single intrarectal dose of 30% ethanol (ETOH group, 100 µl), while the control (CTRL) group received no ethanol. Preventive taVNS (10V, 20Hz & 500us, 10min/day) or no stimulation (clipping only) began one day before ethanol injection and continued for four days. Colonic and splenic levels of interleukin (IL)-10, tumor growth factor (TGF)-β, tumor necrosis factor (TNF)-α, pro-apoptotic markers Bcl-2-associated X protein (BAX), Bcl-2 homologous antagonist killer (BAK1), caspase8, and pro-survival BCL2 associated agonist of cell death (BAD) were measured using qRT-PCR and ELISA.

**Results:**

taVNS significantly increased IL-10 mRNA expression (p<0.0001) and both mRNA (p<0.015) and protein (p<0.0001) levels of TGF-β in CTRL conditions, with no changes in the ETOH group. Mice demonstrated unchanged IL-10 protein levels in the colon in the presence or absence of taVNS. However, in CTRL conditions, taVNS downregulated BAX (p<0.0001), BAK1(p<0.0001), and caspase8 (p<0.0002) mRNA expression significantly, with no effect in the ETOH group. Additionally, BAD (p<0.0001) mRNA expression increased significantly in CTRL conditions but remained unchanged in the ETOH group. In the spleen, taVNS decreased TNF-α (p<0.04) and increased TGF-β (p<0.0007) protein levels in CTRL conditions without impacting the ETOH group.

**Conclusions:**

Our findings demonstrated that taVNS successfully regulated systemic and local cytokines and apoptosis-related markers in control mice. However, a slight irritation induced by ethanol abolished its effects, indicating that ethanol associated with a slight mucosa irritation can modify the taVNS outcome.

**Funding Agencies:**

CIHR

